# Supplementary material for: In-situ physical adjoint computing in multiple-scattering electromagnetic environments for wave control
Source: Nat Commun. 2025 Dec 13;16:11466. doi: 10.1038/s41467-025-66385-5 (PMC12749979; doi:10.1038/s41467-025-66385-5)
Supplement: Supplementary file 1 — Supplementary Information [file 41467_2025_66385_MOESM1_ESM.pdf]

Supplementary Information

In-situ Physical Adjoint Computing in multiple-scattering electromagnetic environments for wave control

John Guillamon[1,†], ChengZhen Wang[1,†], Zin Lin[2], Tsampikos Kottos[1,*]

[1]Wave Transport in Complex Systems Lab, Department of Physics, Wesleyan University, Middletown, CT-06459, USA

[2]Bradley Department of Electrical and Computer Engineering, Virginia Tech, Blacksburg, VA-24060, USA

[†]These authors contributed equally to this work.

[*] Corresponding Author: Tsampikos Kottos; email: tkottos@wesleyan.edu.

Supplementary Note 1. Time-Performance Benchmark

To compare the per-iteration runtime of our in-situ adjoint-based gradient evaluation against an in-situ central finite-difference scheme, we model four platform-dependent latency classes: (i) measurement latency t_m (trigger, acquisition, and on-instrument calibration/processing), (ii) interconnect/propagation latency t_ℓ (host–instrument command/response and data movement), (iii) compute latency t_c (host-side intermediate arithmetic such as objective/adjoint assembly), and (iv) actuation latency t_o (physical parameter updates and settling of the hardware). We denote with p the number of tunable parameters and with $\alpha \geq 1$ the mean number of actuation operations required to apply the update computed at the end of each iteration.

An adjoint iteration consists of: (1) a forward measurement and objective evaluation; (2) an adjoint-field measurement; (3) host-side construction of the gradient; and (4) application of the parameter update. The first three steps incur a constant number of interconnect transactions and measurements independent of p; only the host-side gradient assembly scales with p. This yields

$$t_{\text{adj}}(p) \;=\; 2t_m \;+\; 6t_\ell \;+\; (2+p)\,t_c \;+\; \alpha\, t_o$$

where the constant factors arise from a conservative accounting of round-trip link events for command, acknowledgement, and data flow in steps (1)–(2), plus one actuation block to commit the update in step (4).

By contrast, a central finite-difference iteration must, for each parameter $i \in \{1,\ldots,p\}$, (a) actuate to $x_i + h$, measure $f(x_i + h)$; (b) actuate to $x_i - h$, measure $f(x_i - h)$; and (c) restore the parameter to its nominal value before proceeding. This induces **two** measurements and roughly **three** actuation operations per parameter, each surrounded by a small, constant number of interconnect transactions; the host-side arithmetic remains negligible relative to physical and I/O latencies. Aggregating these costs with the (single) objective re-evaluation and the final update actuation produces

$$t_{\mathrm{FD}}(p) \;=\; t_c \;+\; t_m \;+\; \alpha\, t_o \;+\; 4t_\ell \;+\; p\big(t_c + 2t_m + 3t_o + 10t_\ell\big)$$

where the per-parameter slope reflects the $\pm$ measurements, set/restore moves, and associated link traffic.

We empirically quantified the latency terms on our platform and instantiated the timing model accordingly. Measurement latency t_m was taken from the vendor benchmark of our Keysight P5023B VNA: 6.7 ms for a 1601-point, two-port calibrated sweep, i.e., $\approx$ 4.2 μs per point at the IFBW used here (we will assume a two-port system, for a larger system this will increase the calibration correction time). Interconnect latency t_ℓ is dominated not by cable flight time ($\sim$ 16 ns/m for USB-C) but by host-scheduled USB microframes, so we model a conservative $t_\ell = 125$ μs per command/response transaction. Compute latency t_c on the host, using Numba-optimized Python on an Intel Core i9-14900K, is $\approx$ 3 ns per primitive operation and is negligible relative to I/O and mechanics; we therefore take $t_c = 3$ ns. Actuation latency t_o—the dominant term in practice—is determined by our stepper-motor mechanical updates; after current tuning, our system executes 0.01 mm moves in $\approx$ 0.1 s. A typical update requires a small, fixed number of such micro-moves; we model this with an actuation multiplier $\alpha \approx 10$ averaged from our experimental results, giving $\alpha t_o \approx 1$ s per iteration to commit the new setpoint.

With these platform measurements, the per-iteration runtimes for adjoint and central finite-difference (FD) evaluations were plotted for $p = 1, \dots, 1000$ in Supplementary Fig. 1. Numerically, these values yield a nearly constant adjoint curve, $t_{\mathrm{adj}}(p) \approx 1.0006$ s for $p \le 10^3$, since the only $O(p)$ term is $p\, t_c$ and t_c is nanosecond-scale. In contrast, FD grows linearly with p with per-parameter slope

$$10t_\ell + 2t_m + 3t_o - t_c \;\approx\; 0.001 \text{ s} + 0.000008 \text{ s} + 0.3 \text{ s} \;\approx\; 0.301 \text{ s},$$

so that $t_{\mathrm{FD}}(100) \approx 31.1$ s and $t_{\mathrm{FD}}(1000) \approx 302$ s (~ 5.0 min) per iteration, versus ≈ 1.00 s for adjoint in both cases.

We clearly observe that, per iteration, the FD runtime scales linearly in p with respect to all latency classes—most notably actuation, which incurs three physical operations per parameter—whereas adjoint's only $O(p)$ term is the negligible host arithmetic. Concretely, for each parameter, FD requires two additional measurements, roughly three actuator moves (set $+$, set $-$, restore), and on the order of ten host–instrument link transactions; these physical I/O costs dominate t_c on our platform and are the source of the steep FD slope. In practice, additional guardrails (e.g., verification reads to prevent desynchronization) would introduce further interconnect and measurement events not included here, which would widen the gap in favor of adjoint. Moreover, while our platform is not state-of-the-art, we should expect actuation time to dominate the other forms of latency in general.

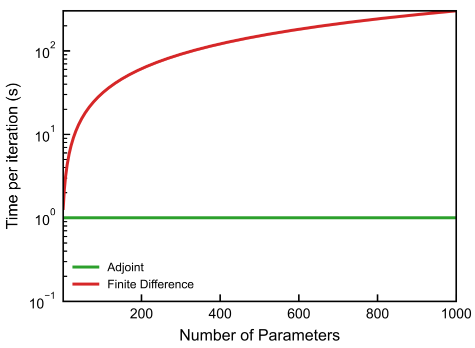

Supplementary Figure 1. Time-Performance Benchmark of iPAC. Per-iteration runtime comparison between in-situ adjoint and central finite-difference methods as a function of parameter count p.

Supplementary Note 2. Performance of Adjoint Optimization Method in Time-Varying Enviroments

All networks have $V = 75$ vertices, with $V_{\text{bulk}} = 50$ of them forming their bulk. The optimization parameters were chosen to be 80% of the bulk bonds. In order to simualte a dynamical environment, a fraction of the remaining (non-tunable) bulk bonds have been randomly varied at each iteration of the Adjoint Optimization process. The length of each of these non-tunable bonds were taken from a uniform distribution $L_b \in [\bar{L_b} - w, \bar{L_b} + w]$ where $\bar{L_b}$ is the initial bond length (which was also taken from a uniform distribution $\bar{L_b} \in [21.2, 28.8]$cm. In our simulations we have enforced the constraint that at each iteration the bond-length variations were summed up to a net zero value.

Across 100 independent random graph trials per objective, we varied w from 10^{-8} to 10cm. For each modality, we selected the connectivity of the graphs based on the highest baseline performance from our large scale simulations: TMT simulations used a connectivity that resulted in a network with topological entropy $h \approx 1$; CPA used a network with $h \approx 3$; Invisibility used a network with $h \approx 2$. Finally, we have repeated the analysis for each of the three tasks for three different fractions of dynamically modulated bonds corresponding to 100% of the non-tunable bulk bonds; 50% of the non-tunable bulk bonds; and 10% of the non-tunable bulk bonds.

The results of the simulations are shown in Supplementary Fig. 2**a-c**. From the data, we conclude that the tolerance of optimization efficiency to bond variations w increases in all three cases as the fraction of non-tunable bulk bonds decreases. Importantly, the optimization efficiency is task-dependent, showing a varying degree of robustness against the bond-variation strength w of the environmental variations. We can identify three regimes for all tasks: the "efficient" regime where all tasks optimize reliably with means in line with near their low-noise optima; onset of performance degradation occurs at the $\sim 1 - 100\mu$m-scale: CPA and Invisibility show the earliest decline ($\sim 1\mu$m) with TMT starting at larger variation strengths ($\sim 100\mu$m). The

low tolerance of the CPA and Invisibility modalities to bond variation strengths (as compared to the TMT task) is attributed to their phase-sensitivity.

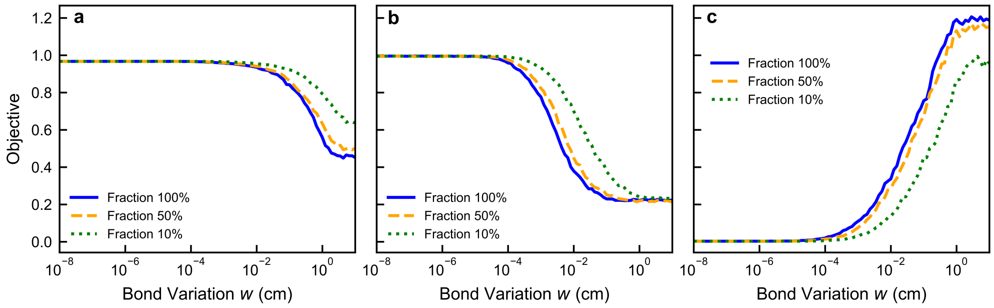

Supplementary Figure 2. Performance of Adjoint Optimization in dynamical environments. Converged value of the objective function versus length variation strength (per iteration) for the three different modalities discussed in the main text: **a** TMT for a network with topological entropy $h \approx 1$; **b** CPA for a network with $h \approx 3$; **c** Invisibility for a network with $h \approx 2$. All networks have $V = 75$ vertices, with $V_{\mathrm{bulk}} = 50$ of them forming its bulk. The optimization parameters were chosen to be $\sigma = 80\%$ of the bulk bonds. Fraction of the remaining (non-tunable) bulk bonds have been radomly changed at every iteration in order to emulate a dynamical environment. Blue solid lines indicate a fraction of 100% of the dynamically modulated bulk bonds; Orange dashed lines indicate a fraction of 50% of the dynamically modulated bulk bonds; Green dotted lines indicate a fraction of 10% of the dynamically modulated bulk bonds.
